# Editorial: Post-Exercise Hypotension: Clinical Applications and Potential Mechanisms

**DOI:** 10.3389/fphys.2022.899497

**Published:** 2022-04-12

**Authors:** Paulo Farinatti, Linda S. Pescatello, Antonio Crisafulli, Redha Taiar, Antonio B. Fernandez

**Affiliations:** ^1^ Laboratory of Physical Activity and Health Promotion, Universidade do Estado do Rio de Janeiro, Rio de Janeiro, Brazil; ^2^ Department of Kinesiology, University of Connecticut, Mansfield, CT, United States; ^3^ Department of Medical Sciences and Public Health, University of Cagliari, Cagliari, Italy; ^4^ Department of Sport Science, Reims University, Reims, France; ^5^ Hartford HealthCare, Hartford, CT, United States

**Keywords:** exercise training, exercise physiology, hypertension, physical activity, health

Professional organizations worldwide recommend exercise training as an essential lifestyle strategy to prevent, treat, and manage elevated blood pressure (BP) (Physical Activity Guidelines Advisory Committee, 2018). Overall, BP reductions of 5–8 mmHg result from exercise training (Day et al.). Additionally, there is evidence that single bouts of exercise can reduce BP compared to control values. This physiological response is termed *postexercise hypotension* (PEH). The known clinical implications of PEH include:

1) PEH occurs immediately in individuals across all levels of physical fitness (Brito et al., 2018);
2) PEH directly correlates with exercise intensity and probably volume (Eicher et al., 2010; Cunha et al., 2016; Fonseca et al., 2018; GjØvaag et al., 2020);
3) Those with the highest BP will experience the largest BP reductions (Pescatello et al., 2019);
4) PEH can persist for up to 24 h, particularly in individuals with elevated BP reducing the need for antihypertensive pharmacotherapy (Pescatello et al., 2019; Day et al.);
5) PEH correlates with the magnitude of BP reductions resulting from exercise training (Hecksteden et al., 2013; Kleinnibbelink et al., 2020) suggesting chronic BP reductions are largely due to PEH. Moreover, PEH may be an easy-to-use predictor for those who respond to exercise training as antihypertensive lifestyle therapy (Wegmann et al., 2018);
6) PEH can be used as a self-monitoring strategy to increase exercise adherence (Zaleski et al., 2019).

The purpose of this Research Topic is to expand upon the growing list of PEH benefits and to provide new evidence on the clinical applications and mechanisms underlying PEH, including the effects of different exercise modalities in different populations; mechanisms of PEH in individuals with normal/high BP; and factors optimizing the PEH response. Nine articles addressing those questions are included, four of them reviews and five original trials. We summarize their major contributions according to the subject categories.

- Methodological Quality of PEH studies

A potential source of bias in PEH studies is the inter-individual variability of the BP responses. Fecchio et al. addressed this question by investigating the inter-individual variation of BP after acute dynamic resistance exercise. PEH showed considerable inter-individual variation and multivariate analyses revealed a greater importance of exercise volume than intensity to optimise the BP reductions.

Day et al. developed an evaluation checklist for PEH studies (PEH√list) based upon contemporary methodological quality standards. The PEH√list contains 38 items divided by sample, study, and intervention characteristics. The authors then conducted a systematic review of aerobic exercise PEH studies and applied the PEH√list. Overall, the items were not well satisfied; especially those with potential confounding effects on PEH. The instrument provides methodological guidance for researchers in future PEH studies.

- PEH response in different populations and associated mechanisms

Barros et al. investigated the effects of moderate intensity acute cycling on BP, arterial function, and heart rate variability in men living with HIV (MLHIV) under antiretroviral therapy. Acute exercise induced PEH in healthy controls, but not in MLHIV. The authors attributed the attenuated PEH in MLHIV to a vascular dysfunction limiting vasodilation.

Iellamo et al. investigated PEH along with hemodynamic and arterial baroreflex mechanisms in patients with coronary artery disease and normal BP. Exercise sessions were 30-min bouts of stationary cycling and calisthenics. Systolic BP was reduced for 12 h *vs*. the non-exercise day, concomitant with a decrease in total peripheral resistance as associated mechanism.

Jarret et al. and coworkers investigated whether obesity modulated PEH after cycling in men with obesity and hypertension. They found the onset of PEH was delayed by an hour or so, however, the magnitude of PEH from 2–4 h postexercise was similar to reports in men without obesity but with hypertension.

The mini-review by Pellinger and Emhoff presented an interesting theoretical approach linking PEH with glucose metabolism. According to the authors, the glucose regulation after exercise might benefit from the sustained postexercise vasodilation often observed in PEH, due to increased macrovascular and microvascular perfusion. This possibility highlights PEH as a desirable effect of regular exercise, particularly in patients with hypertension, diabetes, or metabolic syndrome.

- Effects of different exercise modalities on the PEH

Carpes et al. evaluated the effects of power training *vs.* a non-exercise control session on PEH, BP variability, and endothelial function among older adults with hypertension. BP reductions occurred in the first 60 min postexercise, particularly in men (−15/−7 mmHg *vs.* women), but not during the ambulatory assessment. There were no differences between sexes in BP-variability and endothelial function so the mechanism for these sex differences remain unclear.

Trindade et al. performed a meta-analysis comparing the BP changes after a session of water-based exercise *vs.* control conditions (land exercise or rest) in individuals with hypertension. PEH was most pronounced after aquatic exercise over the nighttime hours.

Marçal et al. performed a meta-analysis comparing the effects of high-intensity interval training (HIIT) and moderate-intensity continuous exercise (MICE) on PEH in individuals with normal and elevated BP. Both exercise protocols were effective in lowering office BP (1–2 mmHg; 30–60 min postexercise). PEH was greater after HIIT than MICE in the ambulatory monitoring over the daytime hours by 5.3/1.6 mmHg.

Overall, those studies reinforce the importance of the subject, expand upon current evidence, and indicate directions for future research:

1) The quality of PEH studies remains a major concern. The standardization of procedures, the inclusion of control comparisons, and the disclosure of baseline BP levels and methods of BP assessment will allow findings to be more trustworthy;
2) Various exercise modalities/protocols elicit PEH. However, confirmation trials investigating these and other modalities/protocols among a variety of populations (obesity, diabetes, heart disease, children, elderly, and paraplegic, etc.) are warranted;
3) The transient effect of acute exercise on BP reinforces that it should be preferably performed daily, but an optimal combination of volume/intensity to induce PEH remains to be determined;
4) A reduction in peripheral resistance seems to be a major determinant of PEH. However, research is needed to determine the relative role of central and peripheral mechanisms, and to clarify the interrelationships between hemodynamic and metabolic responses to acute exercise.
